# Celastrol Prevents Atherosclerosis via Inhibiting LOX-1 and Oxidative Stress

**DOI:** 10.1371/journal.pone.0065477

**Published:** 2013-06-17

**Authors:** Lei Gu, Wenli Bai, Sha Li, Yuqing Zhang, Yi Han, Yue Gu, Guoliang Meng, Liping Xie, Jing Wang, Yujiao Xiao, Liyang Shan, Suming Zhou, Lei Wei, Albert Ferro, Yong Ji

**Affiliations:** 1 State Key Laboratory of Reproductive Medicine, Laboratory of Cardiovascular Disease and Molecular Intervention, Atherosclerosis Research Centre, Nanjing Medical University, Nanjing, China; 2 Department of Cardiology, the Affiliated Jiangning Hospital of Nanjing Medical University, Nanjing, China; 3 Department of Geriatrics, the First Affiliated Hospital of Nanjing Medical University, Nanjing, China; 4 Department of Cardiothoracic Surgery, the First Affiliated Hospital of Nanjing Medical University, Nanjing, China; 5 Department of Clinical Pharmacology, Cardiovascular Division, King's College London, London, United Kingdom; The Chinese University of Hong Kong, Hong Kong

## Abstract

Celastrol is a triterpenoid compound extracted from the Chinese herb Tripterygium wilfordii Hook F. Previous research has revealed its anti-oxidant, anti-inflammatory, anti-cancer and immunosuppressive properties. Here, we investigated whether celastrol inhibits oxidized low-density lipoprotein (oxLDL) induced oxidative stress in RAW 264.7 cells. In addition, the effect of celastrol on atherosclerosis in vivo was assessed in apolipoprotein E knockout (apoE^−/−^) mouse fed a high-fat/high-cholesterol diet (HFC). We found that celastrol significantly attenuated oxLDL-induced excessive expression of lectin-like oxidized low density lipoprotein receptor-1(LOX-1) and generation of reactive oxygen species (ROS) in cultured RAW264.7 macrophages. Celastrol also decreased IκB phosphorylation and degradation and reduced production of inducible nitric oxide synthase (iNOS), nitric oxide (NO) and proinflammatory cytokines such as tumor necrosis factor (TNF)-α and IL-6. Celastrol reduced atherosclerotic plaque size in apoE^−/−^ mice. The expression of LOX-1 within the atherosclerotic lesions and generation of superoxide in mouse aorta were also significantly reduced by celastrol while the lipid profile was not improved. In conclusion, our results show that celastrol inhibits atherosclerotic plaque developing in apoE^−/−^ mice via inhibiting LOX-1 and oxidative stress.

## Introduction

A key determinant of atherosclerotic lesion occurrence is foam cell formation, which is associated with enhanced cholesterol in macrophages [1], and can be elicited by excess oxidized low-density lipoprotein (oxLDL) uptake via scavenger receptors such as lectin-like oxidized low density lipoprotein receptor-1(LOX-1) [2]. LOX-1, a newly-identified vascular receptor for oxLDL, is present on several cell types in the vascular wall, including endothelial cells [2], smooth muscle cells [3] and monocytes/macrophages [4], contributing to the transformation of these cells into foam cells.

Oxidative stress is defined as the imbalanced redox state in which pro-oxidants overwhelm antioxidant capacity, resulting in increased production of reactive oxygen species (ROS). Oxidative stress plays an important role in the pathogenesis of atherosclerosis. ROS have been implicated in the pathogenesis of virtually every stage of vascular lesion formation in atherosclerosis [5]. Traditionally, macrophages have been assumed to be the source of the ROS in the vessel wall, and there is no doubt that these cells play an important role in vessel pathology.

Previous studies showed that ROS can induce the expression of LOX-1. Other studies, stimulation of the endothelial monolayer by binding of oxLDL to LOX-1 produces additional ROS, suggesting a positive feedback loop between ROS and LOX-1 [6], [7].Generators of ROS in macrophages include myeloperoxidase (MPO)-mediated respiratory burst and raft-associated nicotinamide adenine dinucleotide phosphate (NADPH)-oxidase [8]. Uncontrolled ROS production increases oxidative stress and activates key transcription factors including the transcription factors nuclear factor NF-κB, which regulates gene expression for proinflammatory and adhesion molecules [6]. Lipid oxidation through ROS can amplify foam cell formation through oxLDL uptake [8], [9].

Celastrol, a quinine methide triterpenoid isolated from the Chinese herb Tripterygium wilfordii Hook F, exhibits various biological properties, including chemopreventive, antioxidant and neuroprotective effects [10], [11]. Studies about the anti-cancer properties of celastrol showed that celastrol inhibits the growth of estrogen positive human breast cancer cells through modulation of estrogen receptor α [12]. Celastrol has also been proved to be anti-oxidant which can reduce ROS generation, increase heme oxygenase-1 (HO-1) expression and activity in hypertensive rats and vascular smooth muscle cells (VSMCs) [13]. However, the antioxidative effect of celastrol on atherosclerosis has not been investigated.

Mechanistic studies also showed that celastrol suppressed many steps in the induction of inflammation and oxidative stress, including the heat-shock protein 90 and NF-κB signaling pathway [14]. NF-κB is a pleiotropic transcription factor, which has been suggested to play an important role in gene regulation during the oxidative stress and inflammatory that promote atherosclerosis [15], [16].

In our study, we investigated the possible mechanism and effect of celastrol on oxLDL-induced oxidative stress, foam cell formation and atherosclerosis in apolipoprotein E knockout (apoE^−/−^) mice fed with a high-fat/high-cholesterol diet (HFC) and whether the classical NF-κB signal pathway is involved in the antioxidative effect of celastrol.

## Materials and Methods

### Cell culture and materials

Macrophages (RAW 264.7 cells) were purchased from American Type Culture Collection (ATCC, CRL-9609™). Cells were cultured in DMEM with 10% FBS, penicillin (100 U/mL) and streptomycin (100 mg/mL) at 37°C in 5% CO_2_. Confluent cells (85%–90%) were pre-incubated with or without tempol (ROS scavenger, 10 µM to 1000 µM, Sigma) or 1400w (specific iNOS inhibitor, 200 µM, Sigma). Then, cells were stimulated with oxLDL (80 µg/mL, prepared by reaction with CuSO_4_, Yiyuan Biotechnologies, China) and celastrol (25–200 nmol/L, purchased from Calbiochem and was dissolved in DMSO) for 24 hours. A constant concentration of 1% DMSO was maintained in all wells.

### Oil red O staining

Lipid staining was assessed histologically using oil red O. Treated RAW 264.7 cells were incubated with oxLDL (80 µg/mL) in medium containing lipoprotein-deficient human serum for 24 h. Cells were then fixed with 4% w/v paraformaldehyde (30 min, room temperature) and stained with filtered oil red O solution (60 min, room temperature) before microscopic examination (Olympus, Tokyo, Japan).

### Measurement of NO, lipid staining and cholesterol in macrophages

The concentration of NO in culture supernatants was determined as nitrite, a major stable product of NO, by the Griess reagent (1% sulfanilamide in 2.5% H_3_PO_4_ and 0.1% N-[1-naphtyl]ethylenediamine HCl) as described previously. One hundred µl of a culture supernatant was mixed with an equal volume of Griess reagent in a 96 well plate (Becton Dickinson Labware) and incubated for 10 min at room temperature. The absorbance at 540 nm was measured and nitrite concentration was determined using NaNO_2_ as a standard.

Cell lipid was also extracted in order to measure cholesterol esterification (CE). The difference between total cholesterol and free cholesterol is defined as esterified cholesterol [17]. For measurement of cholesterol esterification in cells, cell lipid was extracted in hexane/isopropanol (3∶2, v/v) at room temperature for 30 min and the extract divided into two equal volumes before being dried under nitrogen and re-dissolved in isopropanol. One part was added to buffer (0.05 mmol/L NaH_2_PO_4_ containing 60 U/mL HRP, 0.3 mg/mL 4-hydroxypheyl acetic acid and 0.16 U/mL cholesterol oxidase) and incubated (37°C, 1 h) to measure free cholesterol. The second part was added to an equal volume of the same buffer additional containing 0.5 mmol/L sodium taurocholate, 1% v/v Triton and 0.16 U/mL cholesterol esterase and then incubated (37°C, 2 h) to measure total cholesterol. The difference between the two measurements reflects esterified cholesterol concentration. Fluorescence was detected with an excitation wavelength of 325 nm and an emission wavelength of 415 nm (Biotek SynergyMx Plate reader, USA). The protein pellet was solubilized in 1 mol/L NaOH and protein concentration was determined by the BCA Protein Assay (Thermo Fisher Scientific Inc. IL, USA)

### Measurement of superoxide formation in macrophages

ROS generation was monitored by examining the fluorescence of DHE probe [18]. Cells were treated with oxLDL with or without celastrol for 24 h and then switched to serum-free medium containing DHE (2 µmol/L) further incubated for 60 min and fluorescence was immediately measured by confocal microscopy (Nikon TE2000) equipped with a FITC filter (Ex 480 nm, Em610 nm). Fluorescence intensity was expressed as arbitrary fluorescence units (AU). Data were presented as fold over untreated control group.

### Measurement of glutathione (GSH), glutathione disulfide (GSSG) and myeloperoxidase (MPO) activity in macrophages

Total glutathione (T-GSH), GSH and GSSG were measured spectrophotometrically according to the commercial assay kit procedure (Beyotime Institute of Biotechnology, Nanjing, China). Briefly, T-GSH was assayed using the 5, 5-dithio-bis (2-nitrobenzoic) acid (DTNB)-GSSG reductase recycling. The concentration of reduced GSH in the sample was obtained by subtracting GSSG from T-GSH [19]. MPO activity was assessed by commercial Myeloperoxidase (MPO) Colorimetric Activity Assay Kit (Jancheng Institute of Biotechnology, Nanjing, China) as per the manufacturer's instructions.

### Quantitative real-time PCR (qRT–PCR)

Total RNA was extracted with Trizol and purified using the PureLink RNA mini-kit (Invitrogen, USA). RNA (1 µg) was added as a template to reverse-transcriptase reactions carried out using SuperScript® III First-Strand Synthesis Kit (Invitrogen). Quantitative real-time PCRs (qRT-PCRs) were carried out with the resulting cDNAs in triplicate using iQ SYBR Green Supermix (Biorad) and Biorad CFX384 RealTime System. Experimental Ct values were normalized to 18 s and relative mRNA expression was calculated versus a reference sample. Each sample was run and analyzed in triplicate. The LOX-1 primer sequeces were 5′-GAGCTGCAAACTTTTCAGG-3′ (forward) and reserve: 5′-GTCTTTCATGCAGCAACAG-3′ (reserve). The IL-6 primer sequeces were 5′-GGGAAATCGTGGAAATGAGAAA-3′ (forward) and reserve: 5′-AAGTGCATCATCGTTGTT- CATACA-3′ (reserve). The TNF-α, primer sequeces were 5′-GGCTGCCCCGACTACGT-3′ (forward) and reserve: 5′-TTTCTCCTGGTATGAGATAGCAAATC-3 (reserve).

### Western blot analysis

Cytoplasmic and nuclear protein samples were separated on 10% v/v sodium dodecyl sulfate polyacrylamide gel electrophoresis (SDS-PAGE), transferred onto polyvinylidene fluoride (PVDF) membrane (Millipore, USA) and then immunoblotted with primary anti-NF-κB p65, anti-LOX-1 (1∶1000, Abcam), anti-IκBα, anti-p-IκBα, anti-iNOS, anti-p47-phox(1∶1000, Santa Cruz Biotechnology), anti-α-tublin (1∶1000) or anti-β-actin(1∶2000, Cell Signaling Technology). Protein were visualized by enhanced chemiluminescence substrate (Thermo).

### Animals

Eight-week-old male C57BL/6J mice (obtained from The Laboratory Animal Center of Nanjing Medical University) were used as wild-type (WT) control animals. Twenty-one male eight-week-old apoE^−/−^ mice were purchased from the Animal Center of Beijing University, Beijing, China. All mice were fed a HFC (comprising, wt/wt, 20% protein, 50% carbohydrate, 21% fat, and 0.21% cholesterol) for 4 weeks. ApoE^−/−^ mice were randomly grouped to receive treatment with either celastrol (1 or 2 mg/kg body weight, n = 7, i.p.) or vehicle (10% DMSO, 20% alcohol, 70% PBS, n = 7, i.p.). The dose of celastrol used in this work was based upon prior reports of the effectiveness of this compound in animals with atherosclerosis and tumor [20]–[22]. The C57BL/6J mice were treated with vehicle as well. At the end of experiments, mice were sacrificed and blood samples collected from the abdominal aorta. The blood was mixed with the anti-coagulant Na_2_EDTA (1.5 g/L blood). Plasma was obtained by centrifuging the blood at 800 g at 4°C for 10 min and stored at −80°C until use. The aorta was collected for the lesion analyses. The animal experiments were approved by the Committee on Animal Care of Nanjing Medical University (NJMU-ERLAUA-20100112).

### Measurement of atherosclerotic lesions

Hearts were embedded in tissue optimal cutting temperature (OCT) compound, serially sectioned (5 µm, Leica CM1900 cryostat, Germany), mounted on slides and then stained with oil red O solution for analysis of atherosclerotic lesions as described previously [23]. Total plasma cholesterol (TC), triglycerides (TG), high-density lipoprotein cholesterol (HDL-C) and low-density lipoprotein-cholesterol (LDL-C) concentration were determined using commercially available kits (Zhong Sheng Bei Kong, Peking, China) as per the manufacturer's instructions.

### Immunofluorescence of LOX-1 by confocal microscopy

Cryosections from aortic sinus were prepared as described, which were blocked with 10% donkey serum and incubated with primary antibody against LOX-1 or negative IgG control for 16 h at 4°C. Immunoreactivity was visualized using Alexa Fluor594-conjugated anti-goat IgG (Invitrogen, 1∶1000). The slides were counterstained with DAPI (5 mg/mL, Sigma) and mounted in glycerin jelly medium, then subject to confocal microscopy (LSM710; Carl Zeiss). Three sections per mouse were examined.

### Measurement of superoxide formation in mouse aorta

Superoxide production in tissue sections of mouse aorta was detected by fluorescence microtopography using the fluorescent probe DHE as previously described [18]. For these experiments, aorta was carefully excised and placed in chilled Krebs' buffer. Connective tissue was removed and segments of upper descending thoracic aorta were frozen in OCT compound. Sections (5 µm) were subsequently incubated (30 min, 37°C) in Krebs' HEPES buffer (mmol/L : NaCl 99; KCl 4.7; MgSO_4_ 1.2; KH_2_PO_4_ 1.0; CaCl_2_ 1.9; NaHCO_3_ 25; glucose 11.1, NaHEPES 20, pH 7.4) containing DHE (2 µmol/L) in a light-protected chamber. The slides were examined with a Nikon TE2000 Inverted Microscope (Nikon Ltd., Japan), using excitation and emission wavelengths of 480 and 610 nm respectively.

### Measurement of NF-κB activity, TNF-α, IL-6 and oxLDL levels

NF-κB activity in mouse aorta, concentrations of oxLDL and inflammatory cytokines TNF-α, IL-6 in plasma were determined by enzyme-linked immunosorbent assay (ELISA) according to the manufacturer's instructions (Abcam).

### Statistical analysis

All data are expressed as mean ± standard deviation and were analyzed by one-way ANOVA, followed by Bonferroni comparison. For all the tests, the level of significance was set at *p*<0.05.

## Results

### Celastrol suppresses oxLDL-treated foam cell formation in RAW264.7 cells

**Figure 1:**
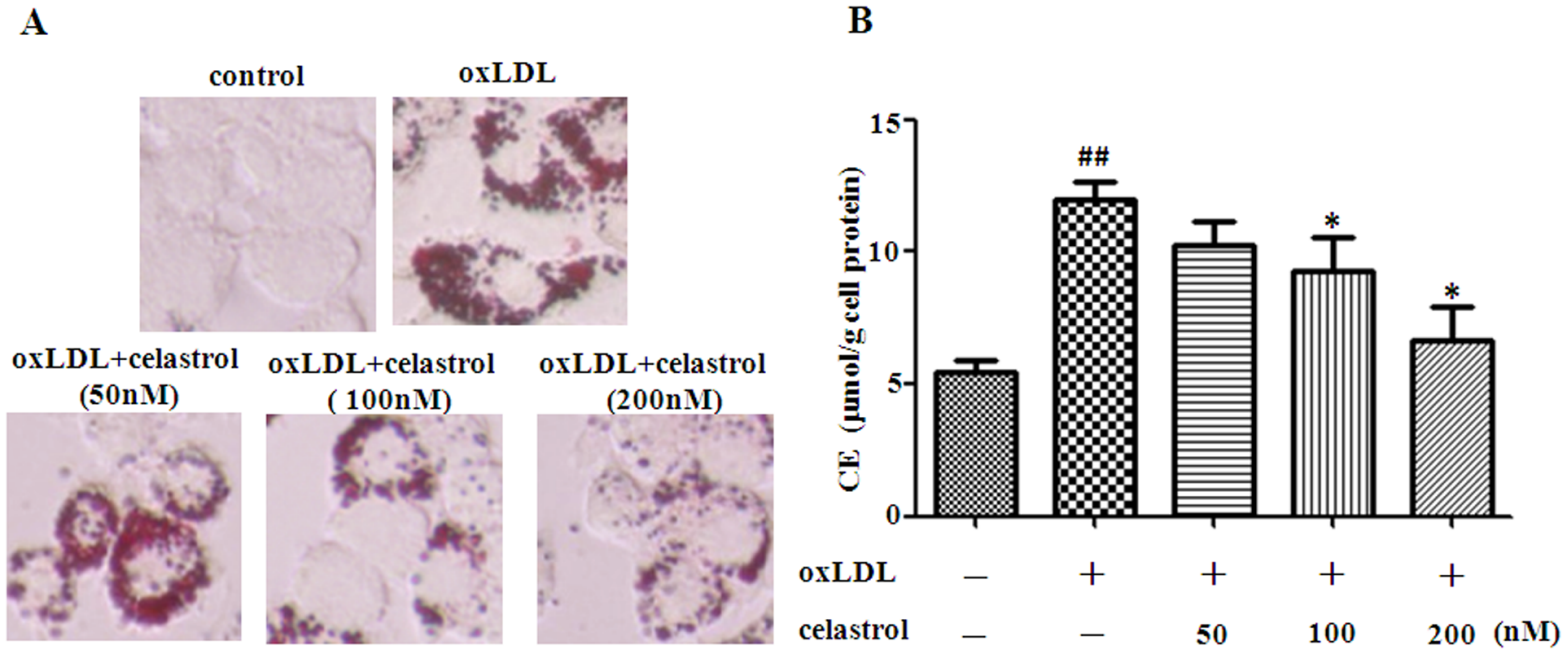
Celastrol reduces oxLDL induced lipid accumulation in RAW264.7 cells. RAW 264.7 cells were exposed to oxLDL (80 µg/mL) in the presence or absence of celastrol (50–200 nM) for 24 h. Representative photographs showing RAW 264.7 cells stained with oil red O (A). Measurement of CE in RAW 264.7 cells (B). ^##^ *p*<0.01, c.f. no treatment,^*^ *p*<0.05, c.f. treatment with oxLDL (n = 5).

The uptake of oxLDL by macrophage induces foam cell formation and promotes the development of atherosclerosis [24]. To determine the effects of celastrol on oxLDL induced foam cell formation, we performed oil red O staining and enzymatic fluorimetry. RAW264.7 macrophages were incubated with oxLDL (80 µg/mL) for 24 h. The addition of oxLDL to the culture medium induced the foam cell formation as the cytoplasmic lipid droplets accumulation was visibly increased (Figure 1A,1B). Both oxLDL induced lipid droplets accumulation and the cellular cholesterol esterification (CE) level were markedly decreased by treatment with celastrol (50–200 nM) (Figure 1A,1B). The results show that celastrol prevents oxLDL induced foam cell formation in RAW264.7 cells.

### Celastrol suppresses oxLDL-induced oxidative stress in RAW264.7 cells

**Figure 2:**
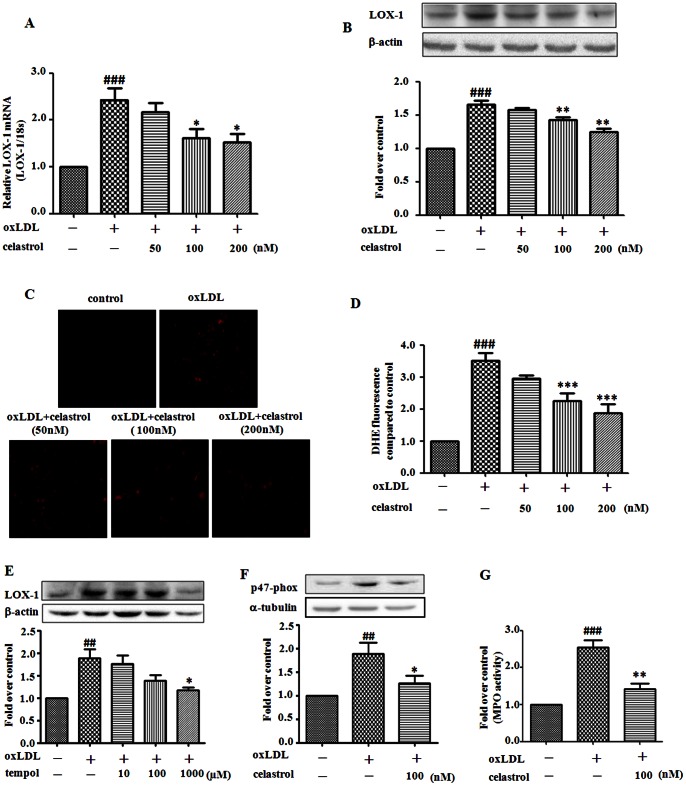
Celastrol inhibits oxidative stress in RAW 264.7 cells induced by oxLDL. RAW 264.7 cells were exposed to oxLDL (80 µg/mL) in the presence or absence of celastrol (50–200 nM) for 24 h. Quantification of LOX-1 mRNA was carried out by real-time PCR (A). Western blot analyses and quantification of LOX-1 protein expression (B). ROS generation monitored by DHE staining (C) and quantification of superoxide production (D). ROS scavenger tempol (10, 100, 1000 µmol/L) and oxLDL (80 µg/mL) were incubated with RAW 264.7 cells for 24 hours. LOX-1 protein expression was analyses and quantification (E). Western blot analyses and quantification of NADPH oxidase p47-phox protein expression (F). Quantification of MPO activity (carried out by a colorimetric activity assay kit) (G). ^##^ *p*<0.01, ^###^ *p*<0.001, c.f. no treatment, ^*^ *p*<0.05, ^**^ *p*<0.01, ^***^ *p*<0.001 c.f. treatment with oxLDL (n = 4–5).

Incubation of RAW 264.7 cells with oxLDL resulted in marked upregulation of LOX-1 expression at both mRNA and protein levels which was inhibited in a concentration-dependent manner by co-treatment with celastrol (50–200 nM) (Figure 2A,2B). Since LOX-1 is a macrophage receptor for oxLDL, the present results suggest that celastrol likely suppresses the uptake of oxLDL by diminishing the expression of the scavenger receptor LOX-1 both on transcription and protein levels.

Previous studies have shown that generation of ROS is associated with LOX-1 expression and activation [6]. We therefore studied ROS generation in response to oxLDL stimulation and its modulation by celastrol in macrophages. As shown in Figure 2C, treatment of macrophages with oxLDL resulted in significant increase in ROS production. Co-treatment with celastrol (50–200 nM) reduced intracellular ROS production in a dose-dependent manner. As shown in Figure 2E, co-treated with oxLDL and ROS scavenger tempol 1 mmol/L [25] inhibited oxLDL induced LOX-1 expression. In Figure 2F and Figure 2G, celastrol inhibited oxLDL induced up-regulation of p47-phox (an important subunit of NADPH oxidase) expression and MPO activity, suggesting celastrol reduced oxidative stress by lowering the activity and expression level of ROS-generating enzyme. Based on our present observation, celastrol might scavenge ROS via reducing the expression and activity of ROS-generating enzyme resulting in LOX-1 level decreasing.

GSSG accumulated and the ratio of GSH to GSSG decreased when cells are exposed to increased levels of oxidative stress. Therefore, the determination of the GSH/GSSG ratio is a useful indicator of oxidative stress in cells and tissues. Cellular levels of GSH and GSSG in Table 1 showed the GSH/GSSG ratio decreased when exposing to oxLDL compared to control, and the ratio significantly increased in oxLDL stimulated macrophages given celastrol (25–100 nM) relative to the oxLDL-only group.

**Table 1:** GSH/GSSG.

	GSH(µmol/L)	GSSG (µmol/L)	GSH/GSSG (ratio)
control	45.02±9.45	1.75±0.24	25.70±3.82
oxLDL	37.00±6.94	2.70±0.98	14.73±4.70###
oxlDL+25 nM CeT	38.69±9.91	1.70±0.48	23.10±3.64*
oxlDL+50 nM CeT	44.70±8.88	1.73±0.42	26.04±2.38***
oxlDL+100 nM CeT	43.69±13.06	1.89±0.63	23.47±1.93**

RAW 264.7 cells were exposed to oxLDL (80 µg/mL) in the presence or absence of celastrol (25–100 nM) for 24 h. The ratio of GSH/GSSG in the cells were measured using commercial kits (A). CeT: celastrol.

### *p*<0.001, c.f. no treatment,

* *p*<0.05,

** *p*<0.01,

*** *p*<0.001 c.f. treatment with oxLDL (n = 5).

### Celastrol exerts its effects by inhibiting NF-κB singnaling pathway in oxLDL-treated RAW264.7 cells

**Figure 3:**
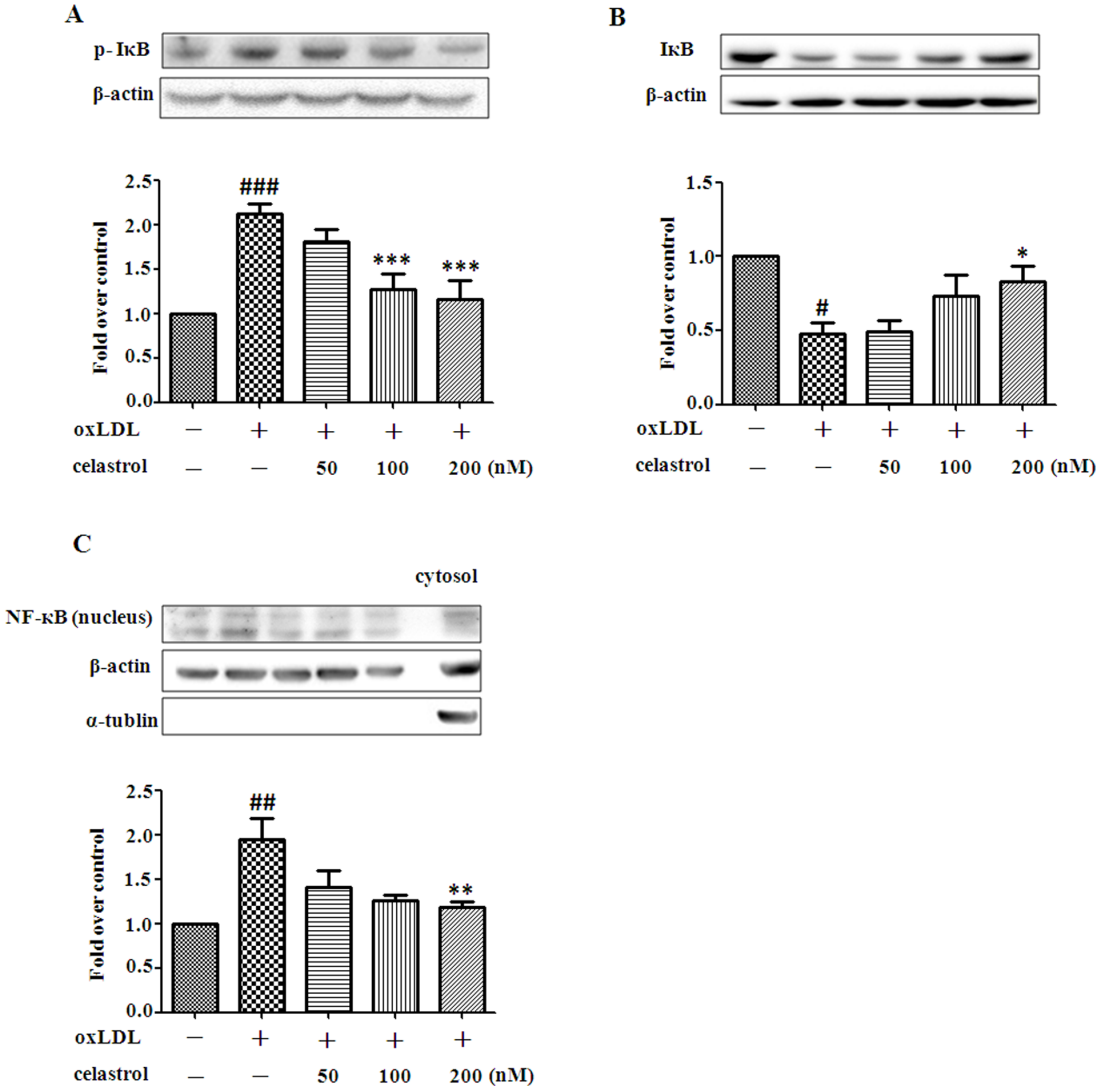
Celastrol suppresses oxidative stress in RAW 264.7 cells by inhibiting NF-κB signaling pathway. RAW 264.7 cells were exposed to oxLDL (80 µg/mL) in the presence or absence of celastrol (50–200 nM) for 24 h. Representative examples of Western blots and quantification of IκBα phosphorylation (A) and IκBα degradation (B). Western blots and quantification of nuclear NF-κB p65 protein expression (C). ^#^ *p*<0.05, ^###^ *p*<0.001 c.f. no treatment, ^*^ *p*<0.05, ^**^ *p*<0.01, ^***^ *p*<0.001 c.f. treatment with oxLDL (n = 4).

To determine the intracellular mechanism under the antioxidative effect of celastrol, we explored the role of redox-sensitive transcription factor NF-κB in this regulatory process. Cytoplasmic protein of IκBα and nuclear protein of NF-κB p65 subunit were determined, respectively. As shown in Figure 3, RAW 264.7 cells challenged with oxLDL showed increased protein expression of nuclear NF-κB p65, IκBα phosphorylation and degradation. Celastrol(50–200 nM) suppressed the increased expression of nuclear NF-κB p65 protein following exposure of RAW 264.7 cells to oxLDL (Figure 3C) as well as IκBα phosphorylation (Figure 3A) and degradation (Figure 3B) .The results suggest that celastrol reduces oxLDL uptake and macrophage foam cell formation by diminishing the expression of LOX-1 via suppression of NF-κB signaling pathway.

### Celastrol exhibits inhibitive effects on oxLDL induced NO production and inflammatory gene expression in RAW264.7 cells

**Figure 4:**
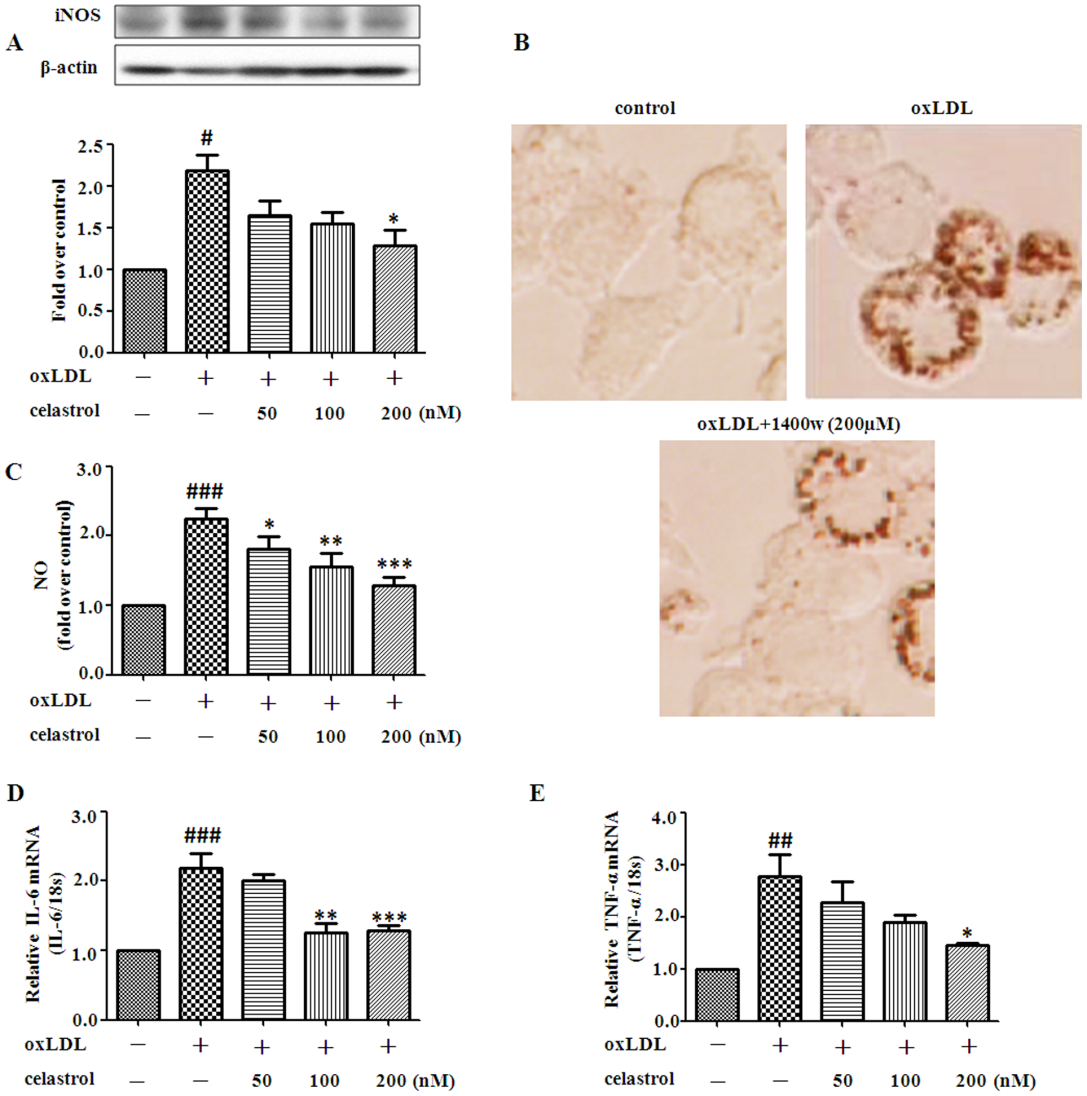
Celastrol inhibits oxLDL induced NO production and inflammatory gene expression in RAW 264.7 cells. RAW 264.7 cells were exposed to oxLDL (80 µg/mL) in the presence or absence of celastrol (50–200 nM) for 24 h. Western blot analysis and quantification of iNOS protein expression (A). The iNOS inhibitor 1400w (200 µmol/L) and oxLDL (80 µg/mL) were incubated with RAW 264.7 cells for 24 hours. Representative photographs showing RAW 264.7 cells stained with oil red O (B). Quantification of NO, IL-6, TNF-α (carried out by real-time PCR )(C–E). ^##^ *p*<0.01, ^###^ *p*<0.001, c.f. no treatment, ^*^ *p*<0.05, ^**^ *p*<0.01, ^***^ *p*<0.001, c.f. treatment with oxLDL (n = 4–5).

NO is quantitatively produced by inducible nitric oxide synthase (iNOS) [26] in response to oxLDL through the activation of NF-κB [27], [28], and that oxLDL induce the expression of genes under the transcriptional control of NF-κB such as TNF-α, IL-6 [29]–[31]. As shown in Figure 4A, incubation of RAW 264.7 cells with oxLDL resulted in marked upregulation of iNOS expression, and it was inhibited in a concentration-dependent mannner by co-treatment with celastrol (50–200 nM). We found iNOS inhibitor 1400w 200 µmol/L [32] prevents lipid accumulation in oxLDL stimulated RAW 264.7 cells as carried out by oil red O staining (Figure 4B), which suggests iNOS plays an important role in foam cell formation, and celastrol might reduced foam cell formation by inhibiting iNOS expression.

Exposure of RAW 264.7 cells to oxLDL also resulted in a significant release of NO, which was determined by measuring the levels of a stable NO metabolite, nitrite, in the culture medium by Griess reaction. Celastrol decreased the oxLDL induced production of NO in a dose-dependent manner (Figure 4C). This agrees with the observed suppression iNOS expression. Celastrol markedly decreased oxLDL induced mRNA expression levels of both IL-6 and TNF-α by qRT-PCR analysis (Figure 4D,4E). These data suggest that celastrol may act as a modulator of the accumulation of inflammatory cytokine production at a transcriptional level.

### Celastrol attenuates atherosclerotic lesion size and aortic superoxide formation in apoE^−/−^ mice

**Figure 5:**
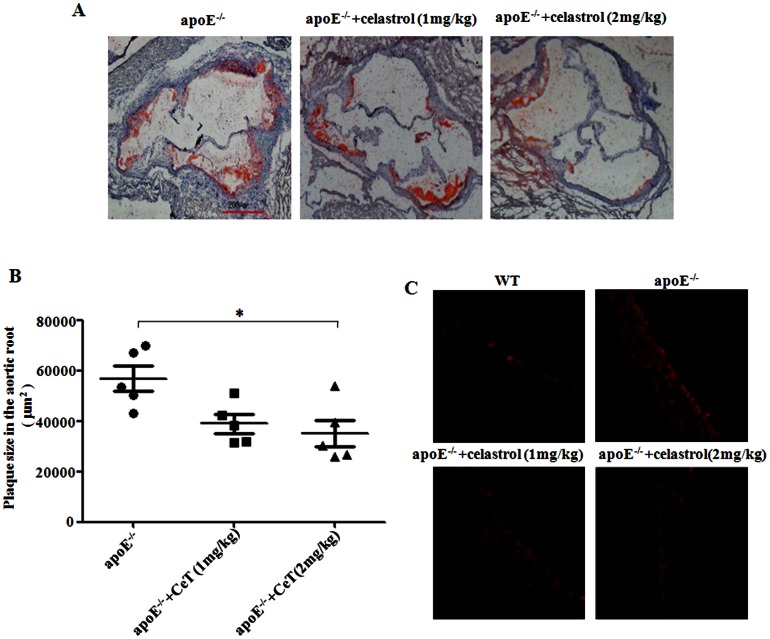
Celastrol attenuates atherosclerotic lesion size and aortic superoxide formation in apoE^−/−^ mice. Celastrol (1 or 2 mg/kg/d, i.p) attenuated atherosclerotic lesion formation. The lesion size was determined by oil red O staining of the aortic sinus (40×magnification) (A). Quantitation of lesion area in oil red O stained aortic sections by Image-Pro Plus software. CeT: celastrol (B). Aortic superoxides were measured by staining aortic root sections with DHE with or without pretreatment with celastrol (C). ^*^ *p*<0.05, c.f. apoE^−/−^ mice (n = 5–8).

To seek in vivo evidence supporting the protective effect of celastrol on atherosclerosis, celastrol was administered (1 or 2 mg/kg body weight, i.p.) to HFC apoE^−/−^ mice. As indicated in Figure 5A, celastrol markedly attenuated atherosclerotic lesion size in aortic root from apoE^−/−^ mice.

To measure the effect of celastrol on aortic superoxide levels, frozen sections from the aortic roots of apoE^−/−^ mice and control mice were stained with DHE with/without pretreatment with celastrol. Celastrol markedly suppressed superoxide levels in the aortic of apoE^−/−^ mice (Figure 5C ).

The lipid profile showed no significant different between apoE^−/−^ mice fed a HFC-diet plus celastrol treatment for 4 weeks and apoE^−/−^ mice without celastrol treatment (Table 2). These results suggest that celastrol reduced atherosclerotic plaque size independent of modulating plasma concentrations of cholesterol and triglyceride.

**Table 2:** Lipid profile.

	TC(mmol/L)	TG (mmol/L)	HDL-C (mmol/L)	LDL-C (mmol/L)
WT	2.59±0.21	0.49±0.20	1.01±0.17	0.77±0.40
apoE^−/−^	9.83±1.18###	1.22±0.41###	0.79±0.16#	2.17±0.84##
apoE^−/−^+1 mg/kg CeT	9.41±0.52	0.87±0.24	0.62±0.16	2.25±0.56
apoE^−/−^+2 mg/kg CeT	8.59±1.21	0.89±0.15	0.73±0.13	2.21±0.55

TC: total cholesterol, TG: triglyceride, HDL-C: high-density lipoprotein-cholesterol, LDL-C: low-density lipoprotein-cholesterol. CeT: celastrol.

# *p*<0.05,

## *p*<0.01,

### *p*<0.001 c.f. WT mice. (n = 6–8).

### Celastrol decreases LOX-1 expression within the atherosclerotic lesions, plasma oxLDL and cytokines levels,aortic NF-κB activity in apoE^−/−^ mice

**Figure 6:**
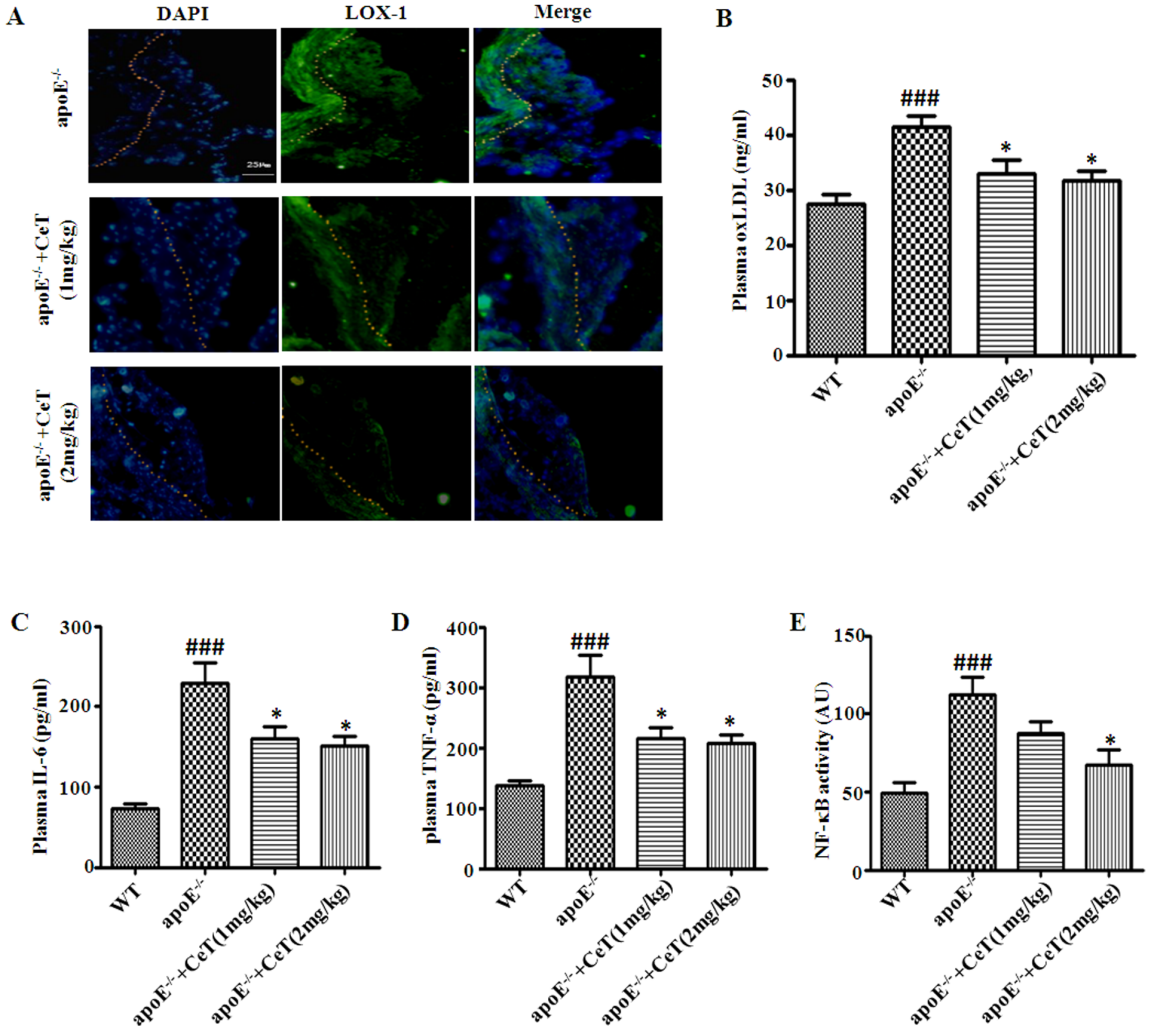
Celastrol decreases LOX-1 expression within the atherosclerotic lesions, plasma oxLDL and cytokines levels, aortic NF-κB activity in apoE^−/−^ mice. Representative cross-sections of aortic root stained with specific antibodies directed against LOX-1 (green), or DAPI (blue). Dotted lines indicate the boundary of lesion and Aortic tunica intima (A). Plasma levels of oxLDL, IL-6, TNF-α and aortic NF-κB activity were determined by ELISA (B–E). CeT: celastrol. ^###^ *p*<0.001, c.f. WT, ^*^ *p*<0.05, c.f. apoE^−/−^ mice (n = 5–8).

As noted previously, celastrol inhibited LOX-1 expression and the generation of a range of pro-inflammatory mediators in RAW 264.7 cells exposed to oxLDL possibly by an effect on the NF-κB pathway. It was thus of interest to determine whether a similar effect might occur in apoE^−/−^ mice administered celastrol. Firstly, confocal microscopy demonstrated that celastrol inhibited LOX-1 expression within the atherosclerotic lesions, while plasma oxLDL level in apoE^−/−^ mice was decreased by celastrol (Figure 6A,6B). Secondly, atherosclerosis in these animals was characterized by a marked increase in plasma TNF-α and IL-6, which upregulation was successfully suppressed by celastrol treatment (Figure 6C,6D). Aortic NF-κB activity was also increased in apoE^−/−^ mice and this effect was also reduced by celastrol treatment (Figure 6E).

## Discussion

Celastrol is a quinone methide triterpenoid isolated from the traditional Chinese medicine “Tunder of God Vine” [33]. Recent studies showed that celastrol is an effective inhibitor of transcription factors and inflammatory cytokines, including NF-κB, IL-1β and TNF-α [14] in LPS- stimulated RAW264.7 cells. Furthermore, celastrol is known to prevent the production of iNOS and lipid peroxidation in rat liver mitochondrial membranes induced by ADP and Fe^2+^ [34], whilst promoting the heat-shock response [33], suggesting that it may have antioxidant properties. The present study evaluates the effect of celastrol on the oxLDL-induced oxidative stress in macrophages.

LOX-1 is an important vascular receptor that mediates oxLDL recognition resulting in foam cell formation [35]. The expression of LOX-1 is increased in atherosclerotic plaques from experimental animals and human atherosclerosis [36]. Recently, LOX-1 knockout mice have also been shown to exhibit markedly reduced atherosclerotic lesions when grown under a high-cholesterol diet [37], suggesting an important role in atherosclerosis. Many anti-atherosclerotic drugs may exert their atheroprotective effects via direct or indirect down-regulation of LOX-1 expression in vascular lesions [38], [39]. Therefore, LOX-1 may be a potential therapeutic target for treating atherogenesis leading to beneficial outcome in vasculature [39], [40].

Our current study demonstrated that celastrol alleviated the lipid accumulation in oxLDL-derived macrophages as revealed by the measurement of the intracellular cholesterol content and oil red O staining. We also found that celastrol reduced the LOX-1 expression both on the mRNA and protein levels. It suggests celastrol can suppress the uptake of oxLDL and thus foam cell formation by diminishing the expression of LOX-1.

ROS are small and highly reactive molecules with important cell signaling roles when maintained at proper cellular concentrations. During times of cell stress ROS levels can greatly increase. Because of their highly reactive nature, ROS can modify other oxygen species, proteins, or lipids, a situation often termed oxidative stress. Previous studies showed that ROS can induce the expression of LOX-1. In other studies, they showed that LOX-1 activation can stimulate ROS generation, suggesting a positive feedback loop between ROS and LOX-1. Indeed, ROS enhances LOX-1 and LOX-1 enhances ROS [6], [7]. In our study, ROS scavenger tempol inhibited oxLDL induced LOX-1 expression, which means which means reducing of ROS might down-regulate LOX-1 expression and inbibiting the positive feedback loop.

Oxidative stress resulted from uncontrolled ROS production has been implicated in the pathogenesis of atherosclerosis. Over the last decades, several studies have examined the potential role of oxidative stress in atherogenesis [41], [42]. Macrophages have a key role in atherosclerotic development and elaborate even more ROS production within the lesion. Previous studies have shown that anti-oxidants reduce NADPH oxidase-mediated ROS production and LOX-1 expression in human macrophages and aortic endothelial cells [43]. Here, we showed that celastrol inhibited oxLDL induced ROS production and LOX-1 expression in macrophages, which suggest the antioxidant activity of celastrol. The results that celastrol inhibited oxLDL induced up-regulation of NADPH oxidase expression and MPO activity showed celastrol reduced oxidative stress by lowering the expression level and activity of ROS-generating enzyme.

Maintaining normal cellular ROS concentrations is vital to the proper physiological function of numerous cell types. An excess production or decreased scavenging of ROS has been implicated in the pathogenesis of atherosclerosis. It has been reported that some thiol compounds, such as GSH and N-acetylcysteine (NAC), can protect cells from oxidative stress by scavenging free radicals and by enzymatic reactions. Glutathione is the most important cellular thiols, modulating redox-regulated signal transduction, acting as a substrate for several peroxidases and other enzymes that prevent or mitigate the deleterious effects of ROS [44], [45]. The ratio of GSH/GSSG in the plasma can reflect changes in the stability of the redox status of an organism [46]. Our results showed that celastrol promoted the GSH redox cycle by raising the intracellular GSH content and GSH/GSSG ratio. In this way, it is part of the mechanism of the anti-oxidative effects of celastrol.

Previous study found that treatment of U937 macrophages with oxLDL increased lipid accumulation as well as intracellular cholesterol content. Overexpression of NF-κB increased, whereas, inhibition of NF-κB expression with siRNA decreased ox-LDL-induced lipid accumulation and cholesterol in macrophages [47]. In human monocytes-derived macrophages, treatment with specific inhibitor for NF-κB (PDTC) attenuated the up-regulation of lipid, cholesterol and triglceride induced by LPS in macrophages [48] It suggested that NF-κB pathway plays an important role in the regulation of foam cell formation.

In the resting state, NF-κB protein is sequestered in the cytosol of the cell by its interactions with the inhibitory protein IκBα. Intracellular signaling associated with oxLDL induce phosphorylation and degradation of IκBα protein, which led free NF-κB that then is translocated to the nucleus where subsequently transactivate the NF-κB-regulated genes like iNOS [49].

There is a certain amount of direct evidence to support the presence of stimulated expression of iNOS in atherosclerosis, which is associated with foam cells. Buttery LD found that immunostaining and in situ hybridization confirmed the presence of iNOS in atherosclerotic vessels, in which it was specifically localized to macrophages and foam cells. Expression of iNOS is associated with atherosclerosis and that the activity of this enzyme under such conditions preferentially promotes the formation and activity of peroxynitrite [50]. This may be important in the pathology of atherosclerosis, which contributes to lipid peroxidation and to vascular damage. We found iNOS inhibitor 1400w prevents foam cell formation as carried out by oil red O staining, which suggests expression of iNOS plays an important role in foam cell formation, and celastrol might reduced foam cell formation by inhibiting iNOS expression.

The molecular mechanisms underlying the anti-atherosclerotic effect of celastrol in macrophages most likely or at least upon inhibition of NF-κB transcription since celastrol reduced the increased expression of nuclear NF-κB and also inhibited IκBα phosphorylation and degradation. These events were associated with increased synthesis of iNOS protein and NO production [51].

Induction of the high-output iNOS usually occurs in an oxidative environment, and thus high levels of NO have the opportunity to react with superoxide leading to peroxynitrite formation. Oxidative stress caused by peroxynitrite can arise from the direct oxidation reactions of peroxynitrous acid or from the formation of oxidizing radicals. Inhibition of excessive iNOS-derived NO production acts against oxidative stress. These data suggest that the protective effects of celastrol against oxLDL-induced oxidative stress in macrophages may include attenuating NO production via down-regulation of iNOS expression.

As previously reported, TNF-α and IL-6 increased intracellular oxLDL accumulation in THP-1/macrophages. This accumulation of intracellular oxLDL induced by TNF-α and IL-6 was concentration-dependent. And TNF-α and IL-6 antibody inhibited intracellular oxLDL accumulation induced by TNF-α and IL-6, respectively. It showed that both TNF-a and IL-6 are not only markers of inflammatory status but also stimulator of foam cell formation [52]. It suggests that the reduction of IL-6 and TNF-α after celastrol treatment plays a role in reducing foam cell formation. In the present study, we showed that celastrol decreased the expression of IL-6 and TNF-α on the mRNA levels, suggesting celastrol improves inflammation at least partially through regulation at the transcriptional level.

To investigate the effect of celastrol on development of atherosclerosis in vivo, we chose the apoE^−/−^ mouse. The apoE^−/−^ mouse is a well-established model to study atherogenesis [53]. ApoE^−/−^ mice have decreased serum apolipoprotein E and exhibit lipid abnormalities and atherosclerosis even on a low-cholesterol diet.

In this study, celastrol was administered (1 or 2 mg/kg body weight, i.p.) to HFC apoE^−/−^ mice. We found that celastrol treatment, 30 days, could reduce atherosclerotic plaque size, consisting with the previous study that celastrol attenuates atherosclerosis in apoE^−/−^ mice by inhibiting inflammation in the arterial wall, but the exact mechanism was not explored [22].

Studies have shown that endothelial LOX-1 overexpression promotes atherogenesis in the common carotid artery of hyperlipidemic apoE^−/−^ mice [54]. Furthermore, LOX-1 transgenic/apoE^−/−^ (LOXtg/apoE^−/−^) mice displayed augmented oxLDL uptake and accelerated inflammatory intramyocardial vasculopathy than control littermates [55]. More importantly, genetic deletion of LOX-1 gene attenuated aortic plaque development in response to high-fat diet in apoE^−/−^ mice. Such a profile suggests that LOX-1 may participate in foam cell formation and that over-expression of LOX-1 might be pro-atherogenic.

Our results showed that celastrol partially abolished LOX-1 expression in atherosclerotic lesions, suggesting its possible mechanism of anti-atherosclerosis relating to the decrease of LOX-1. We also found that superoxide production in vessels of mouse thoracic aorta could be attenuated by celastrol treatment, which indicated that the anti-atherogenic effect of celastrol properly through inhibiting oxidative stress in the arterial wall. In vitro studies showed celastrol inhibited NF-κB and downstream cytokines expression in RAW 264.7 cells exposed to oxLDL. There were similar effects in apoE^−/−^ mice after celastrol administered, which means celastrol reduces atherosclerotic lesions at least in part by inhibiting NF-κB pathway, and shows the anti-inflammation effect of celastrol.

We then checked the lipid profile in mice plasma. The results show that the plasma concentrations of cholesterol, high-density lipoprotein (HDL), low-density lipoprotein (LDL) and triglyceride were not improved significantly by celastrol treatment, indicating that the atheroprotective effects of celastrol were not attributable to elimination of the hypercholesterolemic source in apoE^−/−^ mice.

In conclusion, the present results suggest that pretreatment with celastrol significantly attenuated oxLDL-induced excessive expression of LOX-1 and generation of ROS. We also found that celastrol decreased IκB phosphorylation, NF-κB activation, attenuated excessive production of NO and proinflammatory cytokines such as TNF-α in oxLDL stimulate RAW 264.7 cells. Moreover, we showed that celastrol reduced atherosclerotic plaque size in apoE^−/−^ mice fed a HFC diet by inhibiting oxidative stress, independent of modulating plasma concentrations of cholesterol and triglyceride. Our findings pinpoint a novel mechanism for the anti-atherosclerotic effect of celastrol and suggest that celastrol may potentially be of therapeutic relevance in inhibiting human atherogenesis.
